# Neurosurgical Education Using Cadaver-Free Brain Models and Augmented Reality: First Experiences from a Hands-On Simulation Course for Medical Students

**DOI:** 10.3390/medicina59101791

**Published:** 2023-10-09

**Authors:** Ibrahim E. Efe, Emre Çinkaya, Leonard D. Kuhrt, Melanie M. T. Bruesseler, Armin Mührer-Osmanagic

**Affiliations:** 1 Department of Neurosurgery, Charité—Universitätsmedizin Berlin, Corporate Member of Freie Universität Berlin, Humboldt-Universität zu Berlin, and Berlin Institute of Health, 10117 Berlin, Germany; 2 University Medical Center Hamburg-Eppendorf, 20251 Hamburg, Germany; 3 Facultad de Medicina, Universidad de Sevilla, 41004 Sevilla, Spain; 4 Department of Traumatology and Reconstructive Surgery, Charité—Universitätsmedizin Berlin, Corporate Member of Freie Universität Berlin, Humboldt-Universität zu Berlin, and Berlin Institute of Health, 10117 Berlin, Germany; 5 Faculty of Medicine, Ludwig-Maximilians-University, 80539 Munich, Germany; 6 The GKT School of Medical Education, King’s College London, London WC2R 2LS, UK; 7 Department of Orthopaedics and Neurosurgery, Schulthess Klinik, 8008 Zurich, Switzerland

**Keywords:** UpSurgeOn, augmented reality, 3D printing, neurosurgery education, simulation, medical student

## Abstract

*Background and Objectives*: Neurosurgery has been underrepresented in the medical school curriculum. Advances in augmented reality and 3D printing have opened the way for early practical training through simulations. We assessed the usability of the UpSurgeOn simulation-based training model and report first experiences from a hands-on neurosurgery course for medical students. *Materials and Methods*: We organized a two-day microneurosurgery simulation course tailored to medical students. On day one, three neurosurgeons demonstrated anatomical explorations with the help of life-like physical simulators (BrainBox, UpSurgeOn). The surgical field was projected onto large high-definition screens by a robotic-assisted exoscope (RoboticScope, BHS Technologies). On day two, the students were equipped with microsurgical instruments to explore the surgical anatomy of the pterional, temporal and endoscopic retrosigmoid approaches. With the help of the RoboticScope, they simulated five clipping procedures using the Aneurysm BrainBox. All medical students filled out a digital Likert-scale-based questionnaire to evaluate their experiences. *Results*: Sixteen medical students participated in the course. No medical students had previous experience with UpSurgeOn. All participants agreed that the app helped develop anatomical orientation. They unanimously agreed that this model should be part of residency training. Fourteen out of sixteen students felt that the course solidified their decision to pursue neurosurgery. The same fourteen students rated their learning experience as totally positive, and the remaining two rated it as rather positive. *Conclusions*: The UpSurgeOn educational app and cadaver-free models were perceived as usable and effective tools for the hands-on neuroanatomy and neurosurgery teaching of medical students. Comparative studies may help measure the long-term benefits of UpSurgeOn-assisted teaching over conventional resources.

## 1. Introduction

The growing burden of administrative tasks, restrictions on trainee working hours and higher demands for cost efficiency and patient safety have led to a decrease in operative case volume during residency [1]. Over the past decade, simulation-based surgical training has gained increasing popularity as a means of compensating for reduced operative exposure [2,3,4,5]. Simulators facilitate the acquisition of surgical skills and clinical competencies through the frequent repetition of real-life-like scenarios in a safe and controlled environment [6]. Simulators rely on a wide range of technologies, ranging from three-dimensional (3D) printing to augmented (AR) and virtual reality (VR) systems [2]. VR-based simulators are now available for several cranial and spinal procedures [3,5,7].

The UpSurgeOn psychomotor skill training concept is based on a hybrid (physical and virtual) solution. Users can interact with digital anatomical 3D models through virtual reality and augmented reality. Surgical approaches, including patient positioning, can be simulated in an immersive environment. Craniotomies, dural openings and microanatomical explorations can be performed on physical simulators. Augmented reality can be used to project information or provide guidance on the surgical steps during the simulation [8]. Recent studies have validated the authenticity and effectiveness of UpSurgeOn cadaver-free simulators for residents’ hands-on training [8,9,10]. A recent validation trial showed significant objective skill improvement in neurosurgery residents participating in a 6-week course using the UpSurgeOn psychomotor skill training concept [10]. 

Medical students gain little exposure to neurosurgery within the confines of their schools’ standard curricula. Early exposure, however, lays a crucial foundation for developing a comprehensive impression of the specialty and making informed career decisions [11,12]. Here, we report the feasibility and user experience of UpSurgeOn simulators for medical students’ hands-on teaching. Our primary goal was to familiarize medical students with the anatomy of the most common neurosurgical approaches and to offer them a hands-on microsurgical experience. This hands-on simulation course was the first to utilize the above-mentioned technologies for the neurosurgery education of medical students.

## 2. Materials and Methods

**Figure 1:**
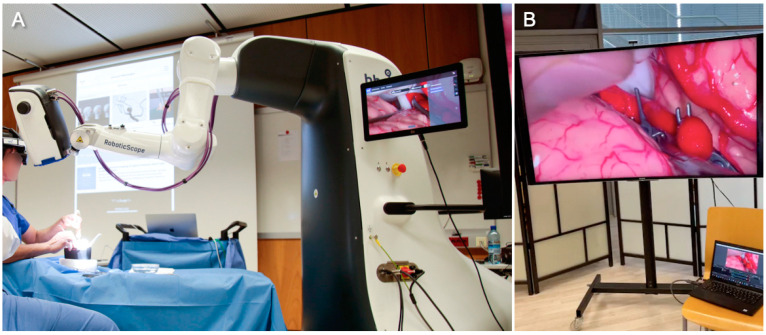
Senior faculty performing a live demonstration of an aneurysm-clipping procedure on a middle cerebral artery bifurcation aneurysm inside the AneurysmBox (UpSurgeOn). The surgical field was magnified and illuminated by the RoboticScope (BHS Technologies), a robotic-assisted exoscope that projects the surgical field into the surgeons’ head-mounted displays (HMDs). The HMD allows hands-free positioning and adjustment of the robotic arm through head movement detection (**A**). On multiple large screens, course participants could follow the surgical steps, including the application of the temporary and permanent clips (**B**).

We organized a free two-day hands-on microneurosurgery simulation course tailored to medical students. A large seminar room of the University of Innsbruck, Austria, served as the course venue. The event was held entirely in English. Each of the two course days consisted of a 4 h academic program. On day one, three senior neurosurgeons (one full professor, two adjunct professors) held academic lectures on the basic principles of neurosurgery using digital three-dimensional models and the augmented reality software of the UpSurgeOn educational app (version 2.0). With the help of the app, they guided the students from clinical decision making to preoperative planning and patient positioning for the most common craniotomies used in neurosurgery. They then demonstrated anatomical explorations and neurosurgical approaches using life-like physical simulators (BrainBox, UpSurgeOn, Milan, Italy). The surgical field was captured by a robotic-assisted exoscope (RoboticScope, BHS Technologies, Innsbruck, Austria) and projected onto multiple large high-definition screens (Figure 1). None of our authors has any affiliations with or involvement in the mentioned entities with any financial or non-financial interests. The authors have not contributed to the development of any of the above-mentioned technologies.

**Figure 2:**
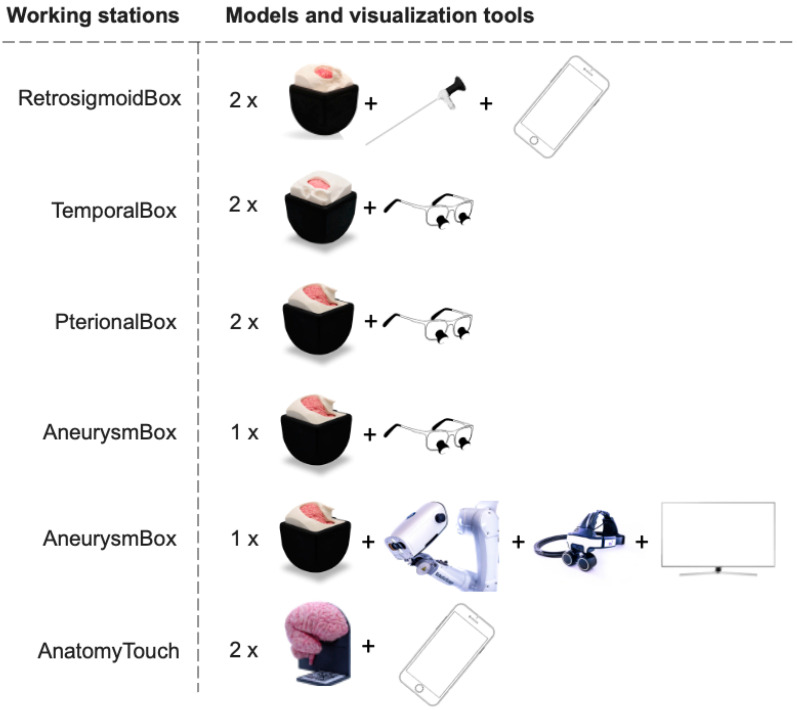
Equipped with surgical loupes or an endoscope, the students rotated through multiple working stations to explore the anatomy of the retrosigmoid, temporal and pterional approaches. The AneurysmBox allowed simulation of a clipping procedure with the assistance of the RoboticScope. Two additional working stations were set up to study neuroanatomy using the AnatomyTouch models and augmented reality features of the UpSurgeOn mobile application.

The senior-most neurosurgeon moderated an interactive panel discussion among the experts. He also encouraged questions from the student audience. Moving into the second day of the program, the students were equipped with precision surgical loupes and an array of microsurgical instruments. They rotated through multiple working stations to become familiar with the anatomical structures encountered in pterional, temporal and endoscopic retrosigmoid approaches (Figure 2).

**Figure 3:**
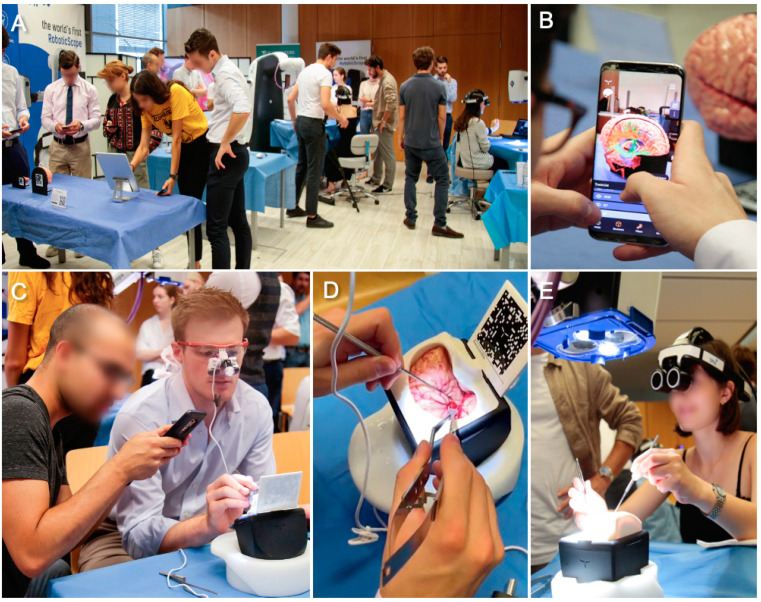
At all working stations, medical students could use the UpSurgeOn mobile app on their smartphones to project anatomical information onto the three-dimensional models via augmented reality (**A**). A student superimposed white matter tracts and the major cerebral blood vessels onto the AnatomyTouch brain hemisphere (**B**). Another participant explored the pterional anatomy using surgical loupes while his colleague helped identify cerebral arteries through augmented reality (**C**). To activate AR-assisted projection of anatomical information, the students simply scanned the BrainBox-specific QR codes with the UpSurgeOn mobile app (**D**). With the help of the RoboticScope, the participants could simulate aneurysm clipping under guidance and high magnification (**E**).

They were further instructed in the handling of the RoboticScope to simulate up to five clipping procedures using the Aneurysm BrainBox. They were supervised by the senior faculty (Figure 3). All students filled out a digital 12-item questionnaire to evaluate the course (Table 1). The primary outcome measure was the students’ subjective overall learning experience. Secondary endpoints included their subjective increase in familiarity with neurosurgical tools and techniques and the courses’ impact on their decision to pursue neurosurgery. 

## 3. Results

**Figure 4:**
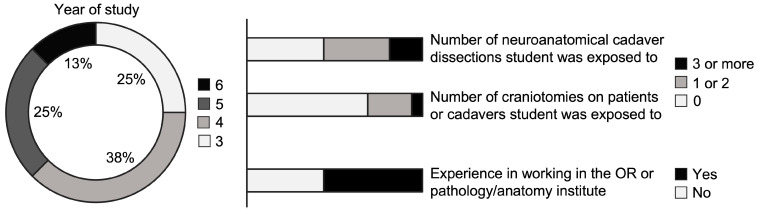
The course participants’ previous experience with cranial surgeries or dissections.

Sixteen medical students from five different countries across Europe participated in the course. All students were in the clinical part of their medical studies. Two were final-year medical students. Only nine students had previously been exposed to a neuroanatomical cadaver dissection at their medical school (Figure 4). No student had previous experience with UpSurgeOn simulators. 

**Figure 5:**
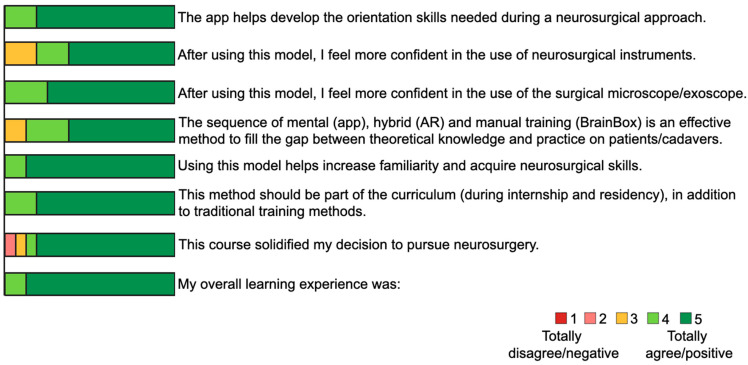
The course participants’ responses to the post-course questionnaire evaluating the quality and educational effectiveness of the UpSurgeOn technology. Questions were designed in the form of a 5-point Likert scale.

All participants had the chance to become familiar with the surgical loupes, the endoscope and the RoboticScope. Two participants could train on only three out of the four different BrainBox scenarios due to lack of time. All students agreed that this model helped them develop neuroanatomical orientation and familiarity with neurosurgical skills. They reported that the use of the RoboticScope helped them gain confidence in the use of a surgical microscope/exoscope. Thirteen students felt more confident in the use of microsurgical instruments. The students unanimously agreed that this model should be part of internship and residency training. Fourteen out of sixteen rated their overall learning experience as totally positive, and the remaining two rated it as rather positive (Figure 5).

## 4. Discussion

Attaining proficiency in neurosurgical skills requires a substantial amount of rigorous training. In light of the challenges posed by diminishing opportunities for operative training, simulation-based training has emerged as a pragmatic solution for surgical residents to compensate for the decrease in operative exposure [1]. Traditionally, surgical simulations relied on the use of cadaveric or animal models [13,14]. However, animal specimens do not compare well to the neurosurgical anatomy of the human brain. Human cadavers are difficult to access and require elaborate preservation and costly maintenance. Further, ethical concerns warrant artificial alternatives. New technologies based on 3D modeling and augmented and virtual reality have helped overcome these limitations [8]. Previous groups have demonstrated the feasibility and usefulness of 3D-printed models of the cerebral vasculature to simulate aneurysm clipping [15,16]. Joseph et al. have developed a sophisticated patient-specific clipping simulator imitating blood circulation and vessel pulsatility [16]. Licci et al. generated an advanced yet low-cost 3D-printed simulator for neuroendoscopic ultrasonic aspirator-assisted tumor resection [17]. Ramirez et al. proposed a free-hand 3D modeling technique using a low-cost commercially available 3D pen to print anatomical structures onto real or artificial bone. Due to a lack of accuracy, their model serves illustrative purposes and should be used only for neuroanatomy teaching but not surgical simulation [18]. 

### 4.1. UpSurgeOn—Product Range and Costs 

UpSurgeOn combines physical and virtual technologies to create a hybrid psychomotor skill training concept. Surgical approaches can be studied using a mobile application. Digital objects can be projected onto the real environment or a physical model. Anatomical explorations and surgical simulations can then be performed on a physical model of the brain [8]. Regardless of the approach, the BrainBox features a black main container placed on top of a white base. These two items are universal and only need to be purchased once as part of a full kit. Additional BrainBox scenarios can hence be purchased at a lower cost without the white base and black box. At the time of this publication, the PterionalBox, RetrosigmoidBox and TemporalBox are available as full kits or scenarios at EUR 799 or EUR 549, respectively. The AneurysmBox can be purchased at EUR 899 or EUR 699, respectively. The UpSurgeOn team has recently expanded its product range to include the InterhemisphericBox and TranssphenoidalBox, as well as several more neurosurgical scenarios that had not yet been available at the time of our hands-on course [19]. Few statistics exist on the affordability of cadaveric specimens. Costs may range from a few hundred dollars to upwards of five thousand dollars, depending on the existing infrastructure and access to body donation programs. In the United States, the estimated average for a single human cadaver is close to USD 2000, excluding delivery and storage costs [20]. 

### 4.2. Previous Validation Studies Using UpSurgeOn Simulators

Petrone et al. organized a similar workshop to ours, featuring the same neurosurgical scenarios (retrosigmoid approach, temporal approach, pterional approach with and without aneurysms). Their workshop was evaluated by 28 residents, however. The residents could practice correct patient positioning and could be trained on the chosen approach in an AR-assisted, step-by-step manner. The authors further timed and evaluated the residents’ surgical performance. Close to 90% of residents found the UpSurgeOn simulators highly realistic and helpful in improving orientation skills in neurosurgery. Among the 28 residents, 24 agreed that this kind of simulation should be part of neurosurgical training [8]. Similarly, all sixteen medical students in our study agreed that this technology should be part of internship and residency training. 

Another group recruited fifteen neurosurgeons to validate the UpSurgeOn Transsphenoidal Box designed to simulate an endoscopic endonasal transsphenoidal approach for the resection of a sellar tumor. As the approach is known to be technically challenging, the authors sought to objectively measure the technical performance of the users. The users were grouped into senior, intermediate and novice surgeons. The authors could prove that intermediate- and expert-level neurosurgeons were more likely to complete the sphenoidotomy successfully compared to their novice colleagues. Overall, all users agreed that the transnasal transsphenoidal scenario demonstrated face, content and construct validity. The addition of neuro-vascular structures and arachnoid mater was suggested to better imitate the real-life intraoperative experience [9]. A recent trial conducted at the Massachusetts General Hospital showed significant improvements in the technical skills of neurosurgery residents who underwent a 6-week UpSurgeOn-based simulation course. Participants who were early in their training showed the greatest learning benefit. The course participants’ technical performance was recorded on video so as to assess their surgical accuracy and operative time during a supraorbital or pterional craniotomy. Specifically, dural opening, suturing, and anatomical identification under the microscope were objectively evaluated. Introducing such objective performance metrics and conducting repeated simulations can significantly benefit surgical training [10]. 

Ahmed et al. conducted a validity trial using the UpSurgeOn AneurysmBox. Face and content validity were assessed through a post-task questionnaire. Construct validity, however, was objectively measured using, among other metrics, the modified Objective Structure Assessment of Technical Skills (mOSATS). They could demonstrate that expert neurosurgeons achieved significantly higher mOSATS scores than their novice colleagues. Their measurements also showed that expert neurosurgeons exerted significantly lower median force than novice neurosurgeons [21]. Williams et al. utilized similar objective outcome measures to validate the RetrosigmoidBox to simulate a retrosigmoid approach to the cerebellopontine angle. Both novice and expert surgeons from the fields of neurosurgery and ear, nose and throat surgery were given the task of identifying the trigeminal nerve in the cerebellopontine angle. Construct validity was assessed by scoring the users’ technical performance on recorded videos. Again, experts scored higher OSATS scores than their novice colleagues [22].

### 4.3. The Role of Medical Student Education in Neurosurgery

The primary goal of orchestrating this hands-on simulation course was to provide early exposure to neurosurgery. Other groups have also organized successful medical student courses in the field of neurosurgery. In response to the inherent limitations in exposure to surgical subspecialties during the preclinical years of medical education, Zuckerman et al. developed a specialized neurosurgery elective course tailored for first- and second-year medical students. Through practical clinical experience and academic discussions led by senior faculty members, students were introduced to both the professional and personal dimensions of a career in neurosurgery. A total of 35 students were enrolled in the elective course, which ran over a span of two years. The course structure encompassed analyses of peer-reviewed articles, student talks and academic lectures by senior faculty members, as well as personal lectures by the faculty, including question-and-answer sessions. Practical hands-on simulations were not included in their course curricula. Nonetheless, their students reported notable shifts in their thoughts and attitudes toward neurosurgery. They were more inclined to consider neurosurgery as a potential career path. Further, they viewed the quality of life among attending physicians more favorably and were more confident that being a neurosurgeon and having a family is feasible [11]. Unfortunately, we did not perform a pre-course evaluation to assess for changes in course participants’ attitudes towards the field. However, most students agreed that they felt more confident about pursuing neurosurgery as a career in our study, too.

Sansosti et al. also analyzed the impact of a hands-on neurosurgery elective course on the thoughts and attitudes of second-year medical students. In their study, a total of 39 students were engaged in a neurosurgery elective course spanning three iterations. Their curriculum included a blend of academic lectures and practical skills lab sessions. Pre- and post-course surveys, as well as weekly quizzes, were distributed to gauge students’ perspectives and knowledge. They, too, reported a notable shift in students’ perspectives of the field after completing the course. The number of students considering neurosurgery as a career has increased significantly. The students’ understanding of neurosurgery has increased substantially, as well [23]. We did not assess our students’ knowledge through pre- and post-course quizzes. We believe that such knowledge assessments could help objectively express the participants’ reported subjective learning benefit. 

Atli et al. conducted an intriguing prospective survey study analyzing the effects of a year-long neurosurgery elective course for medical students with an interactive virtual reality platform. They enrolled twelve second-year medical students. The course included lecture-based learning and problem-based learning methods, as well as a hands-on skills lab and a virtual-reality-based training platform. The use of virtual reality was reported by all course participants to have helped them gain a deeper understanding of both neurosurgery and neuroanatomy. Ninety-two percent further reported that virtual reality helped them better retain the anatomic details of the brain and the spine [24]. 

Recently, Takoutsing et al. reported data from their UpSurgeOn-based neurosurgery and neuroanatomy teaching courses for medical students in Cameroon [25]. This is of particular interest as one of UpSurgeOn’s main objectives is to help bridge the gap between high- and low-and-middle-income countries with respect to educational resources and the quality of training. In resource-challenged environments, standard education programs and high-quality training are often unavailable, owing to the lack of trained professionals and adequate facilities. Digital tools, including the UpSurgeOn psychomotor skill training concept, may help narrow such knowledge and skill gaps [26]. 

Takoutsing et al. included 86 students who participated in UpSurgeOn-based courses across Cameroon. Nearly two-thirds of their students were in their preclinical years. Over 90% of their medical student participants reported having no prior exposure to a neurosurgical cadaver dissection. Interestingly, one-third of their participants had already used the UpSurgeOn system before. Similar to our findings, the majority of students agreed that this tool helped them gain familiarity with neurosurgical skills and develop orientation skills needed during a neurosurgical approach. Also, most medical students felt that this type of education should be part of the standard training curriculum [25]. 

Greuter et al. assessed the effectiveness of 3D virtual reality models in training students on spatial orientation in neurovascular anatomy. Specifically, they enrolled neurosurgical residents and medical students who were given the task of detecting and correctly describing aneurysms on either traditional 2D images or 3D virtual reality models. Participants who used the 3D VR models were shown to detect aneurysms in less time compared to those who used 2D images. While there was no statistically significant difference among residents, medical students benefited significantly from the 3D VR models. The majority of participants preferred the 3D VR models over the traditional 2D images, reporting that virtual reality helped enhance spatial understanding. The authors concluded that the use of virtual reality can be of substantial help in surgical training and preoperative planning of complex procedures [27].

For the reasons stated above, we believe that simulation-based training and extended reality technologies should be made more accessible not only to young trainees but also to medical students considering neurosurgery residency.

### 4.4. RoboticScope

Over the past decade, a large number of robotic technologies have been introduced to surgical visualization [28]. Thanks to the rapid development of continuum robots, the field of endoscopy has been impacted the most [29,30,31]. In our setup, retrosigmoid workstations were equipped with endoscopic cameras connected to a smartphone. The temporal and pterional scenarios could be explored with the help of surgical loupes only. One of the two AneurysmBoxes was magnified and illuminated by the RoboticScope, a high-resolution robotic-assisted 3D exoscope camera system. The RoboticScope is composed of a 3D exoscope coupled to a robotic arm. The surgical field is projected to a head-mounted display (HMD) in real time, allowing optimal surgical ergonomics. While most exoscopes project the surgical field onto large three-dimensional screens, the HMD allows for a visual experience comparable to that of binocular surgical microscopes. A motion sensor within the HMD allows hands-free change in the camera position and angle through the user’s head movements [32,33]. We cannot rule out that the use of the RoboticScope impacted the students’ evaluation of their learning experience and hence their judgment of the UpSurgeOn simulators. This is of particular importance because the aneurysm workstation was considered the most challenging of all scenarios, as students could apply aneurysm clips under high magnification using microsurgical clip applicators.

### 4.5. Limitations of Our Study

The course capacity was intentionally constrained, accommodating a limited cohort of only sixteen students. The small survey sample size rendered robust statistical analyses impossible. Students could only learn hands-on on day two of the course. Only four neurosurgical scenarios (retrosigmoid approach, temporal approach, pterional approach with and without aneurysms) were available for neuroanatomical exploration. The InterhemisphericBox was not available, and the transsphenoidal and suboccipital boxes were still under development at the time of this course. We focused on UpSurgeOn simulators exclusively. Further, comprehensive data were collected through a post-course evaluation survey. It is worth noting that survey items were, in part, derived from UpSurgeOn’s customer and user feedback questionnaire, which typically targets seasoned neurosurgeons and senior trainees. In our case, nearly two-thirds of the participants were only in their third or fourth year of studies. Hence, in contrast to previous studies, we did not evaluate the realism of the brain models, as medical students lack the necessary experience to do so. We did not objectify the students’ knowledge or skill level before and after the course. Pre- and post-course assessments may help objectively express the knowledge gained and improvement in technical skills in future studies. Objective measures such as the Objective Structure Assessment of Technical Skills (OSATS) may serve as useful tools for the evaluation of technical performance.

### 4.6. Outlook

Overall, we believe that simulation-based hands-on courses like this provide a unique learning opportunity to students who would otherwise gain little practical surgical education during their regular medical school curriculum. It may further help them make an informed career decision on whether to pursue a microsurgical discipline. Considering the rising load of administrative tasks and the decline in operative cases, the importance of simulations will likely grow over the next years to ensure adequate training and patient safety. Future studies should assess the benefits of using UpSurgeOn simulators over a longer time span. The impact of education with UpSurgeOn technologies on neuroanatomical knowledge and technical skills could be measured objectively. Comparative trials may help prove the objective benefit of using UpSurgeOn simulators over conventional resources in the future. Further, UpSurgeOn simulators could be compared to those of other companies and research groups to identify the strongest factors for learning benefits. 

## 5. Conclusions

The UpSurgeOn educational app, in combination with the use of real-life-like cadaver-free models, allows an immersive approach to neurosurgery teaching in the form of hands-on simulations. Workshops like this serve as an invaluable tool in providing students with a practical insight into neurosurgery, an experience that would otherwise be inconceivable within the scope of their studies or clinical rotations. Notably, all medical students unanimously rated their overall learning experience as positive. It is worth noting that comparative trials may identify a long-term benefit of UpSurgeOn-assisted teaching methods over conventional approaches in the future.

## Figures and Tables

**Table 1:** All twelve items of the digital post-course questionnaire based on a five-point Likert scale.

1. Which year of study are you in?
2. How many neuroanatomical cadaver dissections have you performed in the past?
3. How many craniotomies have you performed on either cadavers or patients?
4. Have you worked or are you currently working in the operating room, pathology/anatomy institute or any other area dealing with patients or cadavers? If yes, please specify.
5. The Neurosurgery App and the AR simulator help develop neuroanatomical orientation skills needed during a neurosurgical approach.
6. After using this model, I feel more familiar in the use of neurosurgical instruments.
7. After using this model, I feel more familiar in the use of the surgical microscope/exoscope.
8. The sequence of mental training (app), hybrid training (augmented reality) and manual training (BrainBox) is an effective method of training to fill the gap between theoretical knowledge and practice on a real patient/cadaver.
9. Using this model helps to increase familiarity and to acquire neurosurgical skills.
10. This method should be part of the curriculum (during internship and residency), in addition to traditional training methods.
11. This course solidified my decision to pursue neurosurgery.
12. My overall learning experience was:

## Data Availability

The raw data obtained from the digital questionnaire are available on request.
